# Water Intake and Hydration Status among Pregnant Women in the Second Trimester in China: A Three-Day Metabolic Trial

**DOI:** 10.3390/nu16010116

**Published:** 2023-12-29

**Authors:** Zhencheng Xie, Xiaocheng Li, Genyuan Li, Xiaolong Lu, Jieshu Wu, Xiaofang Lin, Yue Yang, Xi Shi, Ye Ding, Zhixu Wang

**Affiliations:** 1 Department of Maternal, Child and Adolescent Health, School of Public Health, Nanjing Medical University, Nanjing 211166, China; zhenchengxie@njmu.edu.cn (Z.X.);; 2 Nanjing Center for Disease Control and Prevention, Nanjing Medical University, Nanjing 210003, China; 3 The Institute of Nutrition and Food Science, Nanjing Medical University, Nanjing 211166, China

**Keywords:** total water intake, hydration biomarkers, adequate intake, pregnant women

## Abstract

Adequate water intake and optimal hydration status during pregnancy are crucial for maternal and infant health. However, research on water intake by pregnant women in China is very limited. This study mainly aimed to observe the daily total water intake (TWI) of pregnant women and its different sources and to investigate the relationship between their water intake and hydration biomarkers. From October to November 2020, a convenience sample of pregnant women in the second trimester (*n* = 21) was recruited. Under conditions close to daily life, they undertook a 3-day metabolic trial. Each participant was provided with sufficient bottled water, and the weight of what they drank each time was measured. The intake of other beverages and foods was measured using a combination of weighing and duplicate portion method. Fasting venous blood and 24 h urine samples were collected and analyzed for the hydration biomarkers, including the serum/urine osmolality, urine pH, urine specific gravity, and the concentrations of major electrolytes in urine and serum. The results showed that the mean daily TWI was 3151 mL, of which water from beverages and foods accounted for 60.1% and 39.9%, respectively. The mean total fluid intake (TFI) was 1970 mL, with plain water being the primary contributor (68.7%, *r* = 0.896). Among the participants, 66.7% (*n* = 14, Group 1) met the TWI recommendation set by the Chinese Nutrition Society. Further analysis revealed that the TFI, water from beverages and foods, plain water, and milk and milk derivatives (MMDs) were significantly higher in Group 1 than those who did not reach the adequate intake value (Group 2) (*p* < 0.05). The results of hydration biomarkers showed that the mean 24 h urine volume in Group 1 was significantly higher than that in Group 2 (*p* < 0.05), while the 24 h urine osmolality, sodium, magnesium, phosphorus, chloride, and creatinine concentrations in Group 1 were significantly lower than those in Group 2 (*p* < 0.05). However, no significant differences were observed in serum biomarkers. Partial correlation analysis showed that TWI was moderately positively correlated with 24 h urine volume (*r =* 0.675) and negatively correlated with urine osmolality, sodium, potassium, magnesium, calcium, phosphorus, and chloride concentrations (*r* = from−0.505 to −0.769), but it was not significantly correlated with serum biomarkers. Therefore, under free-living conditions, increasing the daily intake of plain water and MMDs is beneficial for pregnant women to maintain optimal hydration. The hydration biomarkers in urine are more accurate indicators of water intake and exhibit greater sensitivity compared to serum biomarkers. These findings provide a scientific basis for establishing appropriate water intake and hydration status for pregnant women in China.

## 1. Introduction

Water plays a crucial role in numerous physiological functions in the body [1]. When the amount of water ingested is approximately equal to the amount excreted, the body maintains a state of dynamic water balance. Insufficient or excessive water intake/excretion can cause alterations in the body’s hydration status, potentially harming overall health [2,3,4,5,6]. During the entire pregnancy, the total amount of water in a healthy pregnant woman’s body increases by 6–8 L [7]. This increase is crucial for pregnant women as it helps expand the plasma volume, form the amniotic fluid, and mature the placenta, all of which contribute to creating the appropriate conditions for the healthy growth and optimal development of the fetus [8,9]. Simultaneously, the plasma osmolality threshold that triggers thirst and the secretion of antidiuretic hormones both decreases during pregnancy, allowing mothers to achieve normal water balance in response to these alterations. As pregnancy progresses into its later stages, there is an escalated need for women to consume more food to satisfy burgeoning nutritional and energy requirements. As the vehicle for nutrient metabolism, water participates in the digestion, absorption, circulation, and excretion of nutrients. Therefore, the intake of water and hydration status have significant impacts on the health of both mothers and infants. Although the evidence is limited, it suggests that sufficient water intake during pregnancy can help prevent complications, such as oligohydramnios, constipation, and urinary tract infections [10,11,12], while also reducing the risk of adverse outcomes like gestational hypertension, preterm birth, miscarriage, and low birth weight [13,14]. Furthermore, maintaining adequate hydration has significant positive effects on the neurodevelopment and cognitive function of offspring [15,16]. In summary, the importance of adequate hydration during pregnancy cannot be overstated.

To encourage appropriate water intake by pregnant women, many countries have established adequate intake (AI) values for total water intake (TWI) by pregnant women; however, these values are not unified. For instance, the United States and Canada recommend 3000 mL/day for pregnant women [17], whereas Australia and New Zealand recommend a total of 3100 mL/day. Based on the reference value of the United States, the Chinese Nutrition Society (CNS) has added 300 mL/day to the non-pregnant women’s recommendation and suggested that the AI of TWI for pregnant women be set at 3000 mL/day [18]. However, in different countries, climate, dietary culture, and cultural beliefs are different, which can affect the water requirements. At present, research on the TWI of pregnant women in China for reference is very limited, and each study has its own limitations. The study conducted in Beijing used a semi-quantitative food frequency questionnaire, and the results showed that the average TWI of pregnant women was 2638 mL/day [19]. However, this method investigated the TWI of pregnant women over a long period of time and could not rule out recall bias. Our research group conducted a cross-sectional survey using the online diary with a food atlas, and the results revealed that the median TWI for pregnant women was 2190 mL/day [20]. Although this method improved the accuracy of the data, it was still not comprehensive as it did not consider physical activity and hydration biomarkers. The latest research in 2023 showed that the average water intake of pregnant women in Haikou was 1485 mL/day [21]. Unlike previous two studies, this study used a 7-day 24 h fluid intake questionnaire and evaluate hydration biomarkers in pregnant women’s morning urine and fasting venous blood. However, this study did not take into account the water content in the food. Therefore, it is necessary to adopt more reliable methods to obtain the TWI obtained by pregnant women from beverages and foods, as well as hydration biomarkers in pregnant women’s blood and urine, and comprehensively consider factors such as physical activity and environmental temperature and humidity, in order to provide a basis for recommendations on adequate water intake levels for pregnant women in China.

This study achieved the following goals through a 3-day metabolic trial on women in the second trimester: firstly, to observe the daily TWI and the sources of water intake of pregnant women under free-living conditions; secondly, to explore the relationship between maternal water intake and biomarkers of hydration status; thirdly, to analyze the proportion of pregnant women who meet the AI of TWI for pregnant women as suggested by the CNS and to compare the differences in water intake, proportion from different sources, and biomarkers of hydration status based on whether the recommended threshold is achieved.

## 2. Materials and Methods

### 2.1. Study Participants

From October to November 2020, a convenience sample of pregnant women (*n* = 21) was recruited from Danyang People’s Hospital. Healthy women with singleton pregnancies, aged 20–35 years, currently in the second trimester (13–27 weeks) were eligible for inclusion. This study was approved by the Ethics Committee of Nanjing Medical University (2020–574) and was registered at clinicaltrials.gov as protocol ChiCTR2100044613. Before the study, each participant provided written informed consent.

### 2.2. Study Procedure

**Figure 1:**
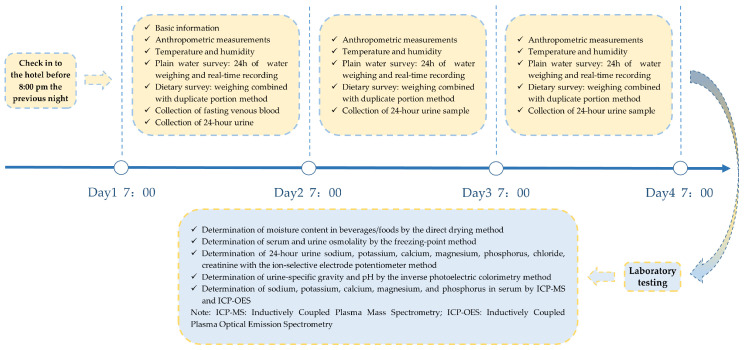
The study procedure.

Under conditions close to daily life, pregnant women undertook a 3-day metabolic trial in a hotel similar to a metabolic ward. Their height, weight, body composition, physical activity, as well as the temperature and humidity of the environment, were all well measured and recorded. We provided participants with sufficient bottled water of the same type (550 mL/bottle, Nongfu Spring, Hangzhou, China) and kept diligent records of the time and amount consumed each time. Meals and snacks were provided buffet-style, with detailed intake records for all foods and beverages using weighing combined with duplicate portion method. Over three days, fasting venous blood and 24 h urine samples from pregnant women were collected for the determination of hydration biomarkers, including serum/urine osmolality, urine pH and urine specific gravity (USG), as well as the levels of major electrolytes in serum/urine (see Figure 1 for more details). As shown in Supplementary Figure S1, strict quality control was implemented at every stage of this study.

### 2.3. Anthropometric Measurements

The age and pre-pregnancy weight of pregnant women were obtained through self-reporting. During the trial, their height and weight were measured by a trained investigator, and the pre-pregnancy body mass index (BMI) and weight gain during pregnancy were calculated. Total body water, intracellular fluid, and extracellular fluid were assessed using bioelectrical impedance analyzer (Model: LEO, 7520, Reo Group, Xuzhou, China) according to the manufacturer’s instructions. A fitness tracker (Model: Xiaomi 3 Standard Edition, Xiaomi, Beijing, China) was used to measure daily steps to reflect the physical activity level of pregnant women.

### 2.4. Temperature and Humidity of the Environment

The temperature and humidity of the indoor and outdoor environments were recorded at 10:00 a.m., 2:00 p.m., and 8:00 p.m. each day for a duration of 3 days. Temperature measurements were accurate to 0.1 °C, while humidity measurements were accurate to within 1% using the JR900A device manufactured by Anymetre in Guangdong, China. In our study, the average indoor and outdoor temperatures were 22.0 °C and 7.4 °C, respectively. The average indoor and outdoor humidity were 32% and 65%, respectively. Detailed data can be found in Supplementary Table S1.

### 2.5. Assessment of the TWI

TWI is the sum of the water from beverages and foods. Beverages mainly included plain water (bottled water provided by our study), fruit and vegetables drinks (FVDs), milk and milk derivatives (MMDs), botanical protein drinks (BPDs), sugar-sweetened drinks (SSDs), and hot beverages (tea and coffee). Foods mainly included staple foods, dishes, porridge, soup, and snacks. These classifications have been described previously [20]. In addition, the total fluid intake (TFI) per day was also added up, which referred to the amount of fluid intake from plain water and beverages.

#### 2.5.1. Water from Plain Water

Throughout the study, each pregnant woman was provided with sufficient bottled water, and the weight of they drank each time was measured using an electronic scale (with an accuracy of 0.1 g). The time and amount of water intake were recorded in detail on the ‘3-day water intake record’.

#### 2.5.2. Water from Foods and Other Beverages

The intakes of beverages other than bottled water and foods were obtained through duplicate portion method. During the 3-day metabolic period, each pregnant women independently selected beverages/foods in the buffet restaurant and was required to take two portions of beverages/foods of the same weight and form (for example, when taking fish, they needed to take two pieces from the same part). One portion was for consumption, and the other portion was for sample collection. After meals, the investigators removed bones and other non-edible parts from each sample, weighed them with an electronic scale (with an accuracy of 0.1 g) and recorded the meal time, beverages/foods name, and actual consumption weight on the ‘3-day dietary intake record’. Subsequently, the sample was placed in a polyethylene storage bag and brought back to the laboratory.

Professional laboratory technicians homogenized the beverages/foods mixture and took two parallel samples. The moisture content in the beverages/foods were measured using the direct drying method, and the actual water intake of the beverages/foods by pregnant women was calculated. In this study, the absolute difference between two independent measurement results obtained under repeatability conditions did not exceed 5% of the mean.

### 2.6. Collection of Urine and Blood Samples

All urine samples from the pregnant women were collected over a 3-day period. Each participant was provided with an electronic scale for weighing the urine (with an accuracy of 0.1 g), a urine pot for collecting urine (with a capacity of 10 L), and a ‘3-day urine sample record’ for recording the time and weight of each urination. The pregnant women urinated in the urine pot every time and then used the electronic scale to measure the weight of urine and recorded it. After each urination, investigators promptly transferred the urine sample to a 4 °C refrigerator. After completing the 24 h urine sample collection, the investigators weighed and recorded the total weight of the urine, and cross-verified it with the recorded value. Then, the investigators thoroughly mixed the 24 h urine sample each day, divided them into equal quantities, and stored them separately in a −80 °C refrigerator.

On the first day of the trial, healthcare professionals collected 2 mL of venous blood from the fasting pregnant women. Then, the blood sample was transported to the laboratory for centrifugation, and the serum was separated and stored in a −80 °C refrigerator.

### 2.7. Measurement of Hydration Biomarkers

Urine and serum osmolality were measured using a molar concentration meter based on osmotic pressure (SMC 30D; Tianhe, Tianjin, China) with the freezing-point method. USG and pH were measured using an automatic urine analyzer (URIT 1600; URIT, Guilin, China) with the inverse photoelectric colorimetry method. Moreover, 24 h urine sodium, potassium, calcium, magnesium, phosphorus, chlorine, and creatinine were measured using an automatic biochemical analyzer with the ion-selective electrode potentiometer method (Cobas 8000; Roche, Basel, Switzerland). The concentrations of sodium, potassium, calcium, magnesium, and phosphorus in serum were determined using inductively coupled plasma optical emission spectroscopy (ICP-OES, ICAP 7200 HS Duo; Thermo Fisher, Waltham, MA, USA) and inductively coupled plasma mass spectrometry (ICP-MS, ICAP RQ; Thermo Fisher, Waltham, MA, USA).

### 2.8. Statistical Analysis

All data were statistically analyzed using the SPSS software package version 26.0 (IBM, New York, NY, USA). Based on the tertiles of TWI, participants were divided into three groups: low TWI level group (L-TWI: 2001–2943 mL), medium TWI level group (M-TWI: 2944–3359 mL) and high TWI level group (H-TWI: 3360–4220 mL). Continuous variables with a normal distribution were presented as mean ± standard deviation (SD), whereas non-normally distributed continuous variables were expressed as median with an interquartile range. Differences among the three groups were evaluated using one-way ANOVA and the Kruskal–Wallis H test (urine pH and USG). After adjusting for potential covariates such as age, gestational weeks, pre-pregnancy BMI, weight gain during pregnancy, and average daily walking steps, a partial correlation was conducted to examine the relationships between TWI, TFI, water from beverages and foods, beverage intake across various categories, and urinary and serum hydration biomarkers.

## 3. Results

### 3.1. Characteristics of the Study Participants

The characteristics of these pregnant women are presented in Table 1. After grouping on the tertiles of TWI, no significant differences were observed in terms of age, height, pre-pregnancy weight, pre-pregnancy BMI, weight gain during pregnancy, total body water, intracellular fluid, extracellular fluid, and daily walking steps (*p* > 0.05).

### 3.2. Daily TWI from Different Sources

The mean daily TWI for the overall population was 3151 mL, of which 1903 mL was from beverages and 1249 mL was from foods, accounting for 60.1% and 39.9% of the TWI, respectively. Further analysis revealed that the mean daily TFI was 1970 mL, with plain water being the primary contributor (68.7%), followed by FVDs (13.5%), MMDs (11.9%), BPDs (4.3%), SSDs (1.5%), and hot beverages (0.1%). The mean daily intakes of these beverages were 1374 mL, 254 mL, 237 mL, 75 mL, 27 mL, and 2 mL, respectively.

As shown in Table 2, the daily TWI and the contribution of water from different sources were compared among different TWI levels, and significant differences were observed in TWI, water from beverages, water from foods, plain water, and BPDs. The results showed that the absolute values of TWI, TFI, water from beverages, water from foods, and plain water in the H-TWI group, as well as the proportion of water from beverages and water from foods to TWI, and the proportion of plain water to TFI were significantly higher than those in the L-TWI group. Conversely, the BPDs in the L-TWI group was significantly higher than that in the H-TWI group (*p* < 0.05).

### 3.3. Comparisons of the Hydration Biomarkers among Different TWI Levels

As shown in Table 3, the mean 24 h urine volume of the total population was 1968 mL. With the increase in daily TWI, the 24 h urine volume increased from the L-TWI group to the H-TWI group, while the 24 h urine osmolality, sodium, phosphorus, and chlorine concentrations gradually decreased, with significant differences among the three groups (*p* < 0.05). However, the concentrations of potassium, calcium, magnesium, and creatinine, USG, and pH in the 24 h urine, as well as the hydration biomarkers in the serum, including serum osmolality, sodium, potassium, calcium, magnesium, and phosphorus concentrations, showed no statistically significant differences among the three groups (*p* > 0.05).

### 3.4. Comparisons of the TWI of Pregnant Women with the AI Value Set by the CNS

As shown in Table 4, the population was divided into two groups according to whether their TWI reached the AI threshold value set by the CNS. The mean daily TWI of the group with the daily TWI reaching the AI value (*n* = 14, Group 1) was 3439 mL, and the contribution of water from beverages was higher than that from foods, with 2094 mL (60.7%) and 1345 mL (39.3%), respectively. The mean daily TWI (2576 mL) of the group whose daily TWI did not reach the AI value (*n* = 7, Group 2) was much lower than that of Group 1, and the contribution of water from beverages (58.9%) was higher than that from foods (41.1%), similar to Group 1. Further analysis revealed that the TFI, water from beverages, water from foods, plain water, and MMDs in Group 1 were significantly higher than those in Group 2, while the intake of BPDs and SSDs showed the opposite trend. The intakes of FVDs and hot beverages were similar between the two groups. The contribution of different sources to TFI was also analyzed. The results showed that the proportions of plain water and MMDs in TFI in Group 1 were higher than those in Group 2, while the proportions of FVDs, BPDs, and SSDs in TFI showed an opposite trend. However, except for the proportion of BPDs to TFI (*p* < 0.05), there was no significant difference in other proportions.

### 3.5. Comparisons of the Hydration Biomarkers with the TWI Levels Set by the CNS

As shown in Table 5, the mean 24 h urine volume of pregnant women in Group 1 was significantly higher than that in Group 2 (2154 mL vs. 1597 mL), while the 24 h urine osmolality, sodium, magnesium, phosphorus, chloride, and creatinine concentrations in Group 1 were significantly lower when compared to Group 2 (*p* < 0.05). There was no statistically significant difference in the hydration biomarkers in the serum between the two groups (*p* > 0.05).

### 3.6. Partial Correlation Analysis

After adjusting for age, gestational week, pre-pregnancy BMI, weight gain during pregnancy, and daily walking steps, the correlations between the water intake from different sources and urine and serum hydration biomarkers are assessed in Table 6. As expected, the TWI showed a strong correlation with TFI, water from beverages, and plain water (*r* = 0.921, 0.922 and 0.856, respectively) and a moderate correlation with water from foods (*r* = 0.653). Further analysis revealed a strong correlation between TFI and plain water (*r* = 0.896). In terms of the urine hydration biomarkers, TWI, TFI, and water from beverages were moderately correlated with 24 h urine volume (*r* = 0.675, 0.689, and 0.697, respectively) and negatively correlated with the 24 h urine osmolality, sodium, potassium, magnesium, calcium, phosphorus, and chloride concentrations (*r* = from −0.505 to −0.769). However, the correlation coefficients between water intake and serum hydration biomarkers, such as serum osmolality, sodium, potassium, calcium, magnesium, and phosphorus concentrations, were low, and no significant correlation was found (*p* > 0.05).

## 4. Discussion

In this study, through a 3-day metabolic trial, we monitored the daily water intake and hydration biomarkers in urine and serum of women in the second trimester under unrestricted conditions, considering factors such as their body composition, environmental temperature and humidity, and daily walking steps. Due to the study’s strict quality control and the most accurate investigation methods for water and dietary intake, as well as 24 h urine collection, this is by far the most reliable study on water intake and hydration status of Chinese pregnant women.

Previous studies on water intake among pregnant women in Mexico [22], Indonesia [23], and Hainan [21] only evaluated TFI and did not consider water from food. In fact, the water in food plays an important role in replenishing the body’s water. Our study comprehensively considered TWI from beverages and food, and the results showed that the daily TWI of pregnant women was 3151 mL, exceeding the data reported in Beijing (2539 mL) and 13 provinces and cities (2190 mL) in China [19,20]. Furthermore, the mean daily intake of water derived from beverages and foods in our study was 1903 mL and 1249 mL, respectively, which also exceeded the data reported in the above two studies. This difference is largely attributed to differences in study methods. Both previous studies estimated the intake of food or beverages and reported them without accurate weighing. Most people tend to estimate the weight of food below the true value [24]. In our study, the intake of plain water was accurately weighed, and the intakes of other beverages and food were obtained through the duplicate portion method, allowing for an accurate assessment of the water intake from different sources by each participant.

We further found that the TWI of 14 pregnant women reached the AI level set by the CNS (3000 mL/day), accounting for 60.7%. This result was more optimistic than previously reported, with 28% of pregnant women in Beijing [19], 16.4% in 13 provinces and cities [20], and 3% in Hainan [21], China. Upon further analysis of the contribution of various sources to TFI, it was revealed that, in addition to plain water, MMDs, which were the second most important source of daily TFI in the group with the daily TWI reaching the AI value, was consumed 141 mL more per day when compared to the group whose daily TWI did not reach the AI value. The trends in the daily intake of BPDs and SSDs were opposite between the two groups. Excessive consumption of BPDs, SSDs, and other beverages by pregnant women can result in increased sugar intake, thereby elevating the risk of developing gestational diabetes and excessive weight gain [25]. Hence, it is advisable to encourage pregnant women to enhance their consumption of plain water, milk, and dairy products while reducing their consumption of sugary beverages in order to guarantee adequate hydration and suitable weight gain during pregnancy.

The regulation of water balance in the body is a complex and dynamic physiological process. Erica Perrier et al. [26] found that the 24 h urine volume and USG respond rapidly to changes in daily water intake and tend to stabilize within 24 h. They also found that the circadian rhythm affects urine volume and USG, with urine volume lower in the morning and evening, and higher in the afternoon. This suggests that collecting 24 h urine for measuring hydration biomarkers may better reflect the relationship between water intake and hydration status. Our research showed that as the TWI of pregnant women increased, their 24 h urine volume also increased, and a substantial positive correlation (*r* = 0.675) existed between them. Simultaneously, there was a significant negative correlation between the TWI of pregnant women and other hydration biomarkers, including urine osmolality, USG, concentrations of sodium, potassium, calcium, phosphorus, chloride, and creatinine, respectively (*r* = from −0.228 to −0.683). This result was comparable to that of McKenzie AL et al. [27] in pregnant women, which mainly focused on the correlation between TFI and urine osmolality, USG, and urine color. The study conducted by Erica Perrier et al. in adults [28] also confirmed that there was a positive correlation between fluid intake and 24 h urine volume (*r* = from 0.74 to 0.79), while there was a negative correlation between fluid intake and 24 h urine osmolality, USG, as well as the concentrations of sodium, potassium, phosphorus, creatinine, urea, and uric acid, respectively (*r* = from −0.49 to −0.73). In the latest study of pregnant women in Haikou [21], it was also found that fluid intake was negatively correlated with morning urine osmolality and USG in pregnant women in Haikou (*r* = from −0.397 to −0.111). However, the correlation coefficient was lower than the results in our study, which may be attributed to the morning urine collected in this study, rather than the 24 h urine.

The biomarkers in blood can also be used to assess the hydration status in the body. However, in our study, there was no significant correlation between serum-related biomarkers and water intake, which was consistent with the results of several previous studies on adults and pregnant women [26,27]. Plasma osmolality mainly comes from crystalline substances dissolved in it, especially electrolytes. It usually remains at a relatively stable level. [17] Osmoreceptors are particularly sensitive to changes in plasma osmolality (as long as they change by 1–2%). If excessive sweating or diarrhea occurs, the plasma osmolality increases, which enhances the stimulation of osmoreceptors, causing an increase in the release of antidiuretic hormones from the neurohypophysis, thereby enhancing the reabsorption of water by the kidneys, reducing urination, retaining water, and restoring plasma osmolality. On the contrary, if a large amount of water is consumed, the plasma osmolality decreases, reducing stimulation of the osmoreceptors, reducing the release of antidiuretic hormones, and causing the kidneys to reabsorb less water, increase urination, and thus expelling excess water [29]. Therefore, plasma osmolality is more suitable as a biomarker for diagnosing acute dehydration [30], and it only increases when water intake is restricted, intense exercise is performed, or the body is exposed to certain environments and loses a large amount of water [31]. In this study, the mean daily walking steps of pregnant women were 5815 steps, with nearly 30 min of moderate-intensity physical activity, and the ambient temperature and humidity were suitable, so the acute dehydration would not occur, and blood biomarkers could remain stable. Based on the above analysis, we believe that under free-living conditions, biomarkers in urine are more sensitive to water intake than those in blood, and urine biomarkers are more suitable as clinical indicators for evaluating water intake of pregnant women.

This study has some limitations to consider. Firstly, as an exploratory study, we wanted to measure the amount of water intake and excretion in pregnant women as accurately as possible. For example, the duplicate portion method used in this study is the most accurate method for assessing dietary intake and reflects the actual amount of water ingested through food. However, this also requires a significant investment of human, material, and financial resources, making it impossible to conduct large-scale studies. Therefore, the sample size of our study was relatively small. Furthermore, although our study monitored the temperature and humidity of the environment, it was conducted at a specific time of the year (from October to November), which means that the study did not take into account the impact of seasonality. Finally, given the operability, this study only investigated women in the second trimester of pregnancy.

## 5. Conclusions

This study obtained accurate data on the water intake of Chinese pregnant women and delved into the relationship between water intake and hydration biomarkers in this population. The findings revealed that 33.3% of pregnant women still had a TWI below the recommended level, and increasing the daily intake of plain water and MMDs for this group is the most effective way to achieve adequate water intake. To evaluate the hydration status of pregnant women, hydration biomarkers in urine can be selected, such as the 24 h urine volume, urine osmolality, and urine electrolyte concentration, which exhibit higher sensitivity compared to serum biomarkers. We hope that these findings can provide clues for developing adequate water intake for Chinese pregnant women. As there are considerable variations in the physiological status of pregnant women at different stages of pregnancy, in the future, it is necessary to carry out studies involving a larger sample size of participants at different stages of pregnancy to monitor their water intake and hydration status. Furthermore, longitudinal monitoring of pregnancy outcomes and infant health is also necessary to observe the long-term effects of maternal hydration status.

## Figures and Tables

**Table 1:** The characteristics of participants.

Variables	Total (*n* = 21)	L-TWI (*n* = 7)	M-TWI (*n* = 7)	H-TWI (*n* = 7)	*p*
Age (years)	28.1 ± 3.0	26.7 ± 3.3	28.9 ± 2.3	28.9 ± 3.3	0.329
Height (cm)	161.4 ± 5.6	162.1 ± 5.4	158.3 ± 2.4	163.7 ± 5.6	0.112
Pre-pregnancy weight (kg)	56.9 ± 7.1	55.9 ± 7.3	57.3 ± 10.3	57.6 ± 3.1	0.909
Pre-pregnancy BMI (kg/m^2^)	21.9 ± 3.0	21.3 ± 3.0	22.9 ± 4.2	21.5 ± 1.5	0.601
Weight gain during pregnancy (kg)	7.0 ± 3.9	6.9 ± 2.3	4.6 ± 2.7	9.5 ± 5.1	0.063
Total body water (kg)	29.6 ± 2.5	29.3 ± 2.5	28.7 ± 2.0	30.9 ± 2.7	0.283
Intracellular fluid (kg)	18.4 ± 1.6	18.2 ± 1.6	17.8 ± 1.3	19.2 ± 1.7	0.251
Extracellular fluid (kg)	11.2 ± 0.9	11.1 ± 1.0	10.8 ± 0.7	11.7 ± 1.0	0.245
Walking steps (steps/day)	5815 ± 1946	5955 ± 1860	4912 ± 1643	6577 ± 2192	0.246

Values are shown as the mean ± standard deviation (SD); BMI: body mass index; L-TWI: low total water intake group; M-TWI: medium total water intake group; H-TWI: high total water intake group.

**Table 2:** The daily water intake (mL) and the contribution of water from different sources (%) among different levels of total water intake.

Variables	Total (*n* = 21)		L-TWI (*n* = 7)		M-TWI (*n* = 7)		H-TWI (*n* = 7)		*p*1	*p*2
	Mean ± SD	%	Mean ± SD	%	Mean ± SD	%	Mean ± SD	%		
Total water intake	3151 ± 524	–	2576 ± 320 ^a^	–	3189 ± 109 ^b^	–	3689 ± 277 ^c^	–	0.001	–
Water from beverages	1903 ± 411	60.1	1521 ± 259 ^a^	58.9	1885 ± 216 ^b^	59.0	2303 ± 303 ^c^	62.3	0.001	0.412
Water from foods	1249 ± 219	39.9	1055 ± 145 ^a^	41.1	1304 ± 158	41.0	1386 ± 212 ^c^	37.7	0.006	0.412
Total fluid intake	1970 ± 415	–	1586 ± 258 ^a^	–	1954 ± 225 ^b^	–	2369 ± 313 ^c^	–	0.001	–
Plain water	1374 ± 426	68.7	1000 ± 345 ^a^	62.1	1312 ± 189 ^b^	67.4	1811 ± 243 ^c^	76.7 ^c^	0.001	0.041
FVDs	254 ± 143	13.5	255 ± 119	16.9	311 ± 190	15.4	197 ± 102	8.2	0.343	0.100
MMDs	237 ± 149	11.9	143 ± 83	9.4	276 ± 157	14.1	292 ± 162	12.1	0.116	0.457
BPDs	75 ± 74	4.3	136 ± 70 ^a^	8.4 ^a^	51 ± 77	2.8	37 ± 30 ^c^	1.7 ^c^	0.017	0.004
SSDs	27 ± 65	1.5	52 ± 105	3.1	5 ± 13	0.3	25 ± 42	1.0	0.423	0.323
Hot beverages	2 ± 10	0.1	0 ± 0	0	0 ± 0	0	7 ± 17	0.3	0.387	0.387

SD: standard deviation; %: calculated by average; MMDs: milk and milk derivatives; FVDs: fruit and vegetables drinks; BPDs: botanical protein drinks; SSDs: sugar-sweetened drinks; L-TWI: low total water intake group; M-TWI: medium total water intake group; H-TWI: high total water intake group. *p*1: statistical values comparing differences in the daily water intake. *p*2: statistical values comparing differences in the contribution of water from different sources. ^a^ statistically significant difference between the L-TWI and M-TWI groups, *p* < 0.05. ^b^ statistically significant difference between the M-TWI and H-TWI groups, *p* < 0.05. ^c^ statistically significant difference between the L-TWI and H-TWI groups, *p* < 0.05.

**Table 3:** The concentrations of urine and serum biomarkers among pregnant women in different levels of total water intake.

Variables	Total (*n* = 21)	L-TWI (*n* = 7)	M-TWI (*n* = 7)	H-TWI (*n* = 7)	*p*
24 h urine					
Volume (mL)	1968 ± 454	1597 ± 306	1909 ± 331 ^b^	2398 ± 321 ^c^	0.001
Osmolality (mOsm/kg)	469 ± 117	569 ± 99 ^a^	457 ± 110	381 ± 55 ^c^	0.004
Sodium (mmol/L)	82.47 ± 19.19	94.78 ± 14.86	82.57 ± 22.56	70.07 ± 11.91 ^c^	0.046
Potassium (mmol/L)	24.97 ± 6.07	28.46 ± 4.64	25.34 ± 6.75	21.12 ± 4.90	0.068
Calcium (mmol/L)	2.94 ± 0.80	3.14 ± 0.77	2.92 ± 0.71	2.75 ± 0.97	0.678
Magnesium (mmol/L)	1.40 ± 0.66	1.65 ± 0.65	1.21 ± 0.45	1.36 ± 0.84	0.465
Phosphorus (mmol/L)	11.20 ± 2.82	13.49 ± 2.62 ^a^	10.59 ± 2.48	9.53 ± 1.95 ^c^	0.016
Chlorine (mmol/L)	58.11 ± 13.89	67.42 ± 10.93	57.92 ± 15.20	48.98 ± 9.70 ^c^	0.036
Creatinine (µmol/L)	4086 ± 1067	4726 ± 917	4092 ± 1235	3440 ± 680	0.071
USG ^&^	1.015 ± 0	1.015 ± 0	1.015 ± 0	1.015 ± 0	0.350
pH ^&^	7.0 ± 0.3	7.0 ± 0.3	7.0 ± 0.2	7.0 ± 0.2	0.817
Serum					
Osmolality (mOsm/kg)	289 ± 4	288 ± 3	290 ± 6	288 ± 3	0.489
Sodium (mmol/L)	119.65 ± 7.77	118.94 ± 7.97	117.48 ± 9.93	122.52 ± 4.85	0.482
Potassium (mmol/L)	6.86 ± 0.64	7.08 ± 0.85	6.77 ± 0.55	6.74 ± 0.52	0.573
Calcium (mmol/L)	2.71 ± 1.27	2.39 ± 0.80	2.86 ± 1.69	2.87 ± 1.31	0.740
Magnesium (mmol/L)	0.64 ± 0.10	0.60 ± 0.08	0.65 ± 0.13	0.68 ± 0.07	0.327
Phosphorus (mmol/L)	4.07 ± 0.51	4.05 ± 0.60	3.77 ± 0.43	4.39 ± 0.32	0.067

USG: urine specific gravity. The formula for calculating 24 h urine volume (mL) = 24 h urine weight (g)/USG. Values are shown as the mean ± standard deviation (SD) unless otherwise specific; ^&^ the data are presented by median ± interquartile range. ^a^ statistically significant difference between the L-TWI and M-TWI groups, *p* < 0.05. ^b^ statistically significant difference between the M-TWI and H-TWI groups, *p* < 0.05. ^c^ statistically significant difference between the L-TWI and H-TWI groups, *p* < 0.05.

**Table 4:** The daily water intake (mL/day) and contribution of water intake from different sources (%) between pregnant women categorized by adequate intakes of total water intake set by the Chinese Nutrition Society.

Variables	Group 1 (*n* = 14, 66.7%)		Group 2 (*n* = 7, 33.3%)		*p*1	*p*2
	Mean ± SD	%	Mean ± SD	%		
Total water intake	3439 ± 329	-	2576 ± 320	-	0.001	–
Water from beverages	2094 ± 333	60.7	1521 ± 259	58.9	0.001	0.483
Water from foods	1345 ± 185	39.3	1055 ± 145	41.1	0.002	0.483
Total fluid intake	2162 ± 339	-	1586 ± 258	-	0.001	-
Plain water	1561 ± 333	72.1	1000 ± 345	62.1	0.002	0.055
FVDs	254 ± 158	11.8	255 ± 119	16.9	0.989	0.183
MMDs	284 ± 154	13.1	143 ± 83	9.4	0.013	0.249
BPDs	44 ± 56	2.3	136 ± 70	8.4	0.004	0.001
SSDs	15 ± 32	0.6	52 ± 105	3.1	0.393	0.310
Hot beverages	3 ± 12	0.2	0 ± 0	0	0.494	0.494

SD: standard deviation; %: calculated by average; TWI: total water intake; AI: adequate intake; CNS: Chinese Nutrition Society; MMDs: milk and milk derivatives; FVDs: fruit and vegetables drinks; BPDs: botanical protein drinks; SSDs: sugar-sweetened drinks. Group 1: the group whose daily TWI reached the AI value set by CNS; Group 2: the group whose daily TWI did not reach the AI value set by CNS; *p*1: statistical values comparing differences in the daily water intake. *p*2: statistical values comparing differences in the contribution of water from different sources.

**Table 5:** The levels of hydration biomarkers in urine and serum between pregnant women categorized by adequate intakes of total water intake set by the Chinese Nutrition Society.

Variables	Group 1 (*n* = 14, 66.7%)	Group 2 (*n* = 7, 33.3%)	*p*
24 h urine			
Volume (mL)	2154 ± 403	1597 ± 306	0.005
Osmolality (mOsm/kg)	419 ± 93	569 ± 99	0.003
Sodium (mmol/L)	76.32 ± 18.50	94.78 ± 14.86	0.034
Potassium (mmol/L)	23.23 ± 6.07	28.46 ± 4.64	0.060
Calcium (mmol/L)	2.84 ± 0.82	3.14 ± 0.77	0.423
Magnesium (mmol/L)	1.28 ± 0.65	1.65 ± 0.65	0.237
Phosphorus (mmol/L)	10.06 ± 2.21	13.49 ± 2.62	0.005
Chlorine (mmol/L)	53.45 ± 13.10	67.42 ± 10.93	0.026
Creatinine (µmol/L)	3766 ± 1016	4726 ± 917	0.049
USG ^&^	1.015 ± 0	1.015 ± 0	0.124
pH ^&^	7.0 ± 0.2	7.0 ± 0.3	0.717
Serum			
Osmolality (mOsm/kg)	289 ± 5	288 ± 3	0.480
Sodium (mmol/L)	120.00 ± 7.95	118.94 ± 7.97	0.777
Potassium (mmol/L)	6.76 ± 0.52	7.08 ± 0.85	0.287
Calcium (mmol/L)	2.87 ± 1.45	2.39 ± 0.80	0.432
Magnesium (mmol/L)	0.67 ± 0.10	0.60 ± 0.08	0.161
Phosphorus (mmol/L)	4.08 ± 0.49	4.05 ± 0.60	0.899

USG: urine specific gravity. The formula for calculating 24 h urine volume (mL) = 24 h urine weight (g)/USG. Values are shown as the mean ± standard deviation (SD) unless otherwise specified; ^&^ the data are presented by median ± interquartile range; Group 1: the group whose daily TWI reached the AI value set by CNS; Group 2: the group whose daily TWI did not reach the AI value set by CNS.

**Table 6:** Partial correlation between the water intake from different sources and urine and serum hydration biomarkers adjusted for age, gestational weeks, pre-pregnancy BMI, weight gain during pregnancy, and daily walking steps.

Variables	Total Water Intake	Water from Beverages	Water from Foods	Total Fluid Intake
Total water intake(mL)	1.000	0.922 ***	0.653 **	0.921 ***
Water from beverages (mL)	0.922 ***	1.000	0.308	0.999 ***
Water from foods (mL)	0.653 **	0.308	1.000	0.309
Total fluid intake (mL)	0.921 ***	0.999 ***	0.309	1.000
Plain water (mL)	0.856 ***	0.913 ***	0.317	0.896 ***
FVDs (mL)	0.025	0.171	–0.272	0.185
MMDs (mL)	0.423	0.412	0.233	0.442
BPDs (mL)	–0.411	–0.494	–0.044	–0.495
SSDs (mL)	0.107	0.073	0.120	0.096
Hot beverages (mL)	0.043	–0.046	0.194	–0.032
24 h urine				
Volume (mL)	0.675 **	0.697 **	0.294	0.689 **
Osmolality (mOsm/kg)	–0.683 **	–0.769 ***	–0.174	–0.767 ***
Sodium (mmol/L)	–0.554 *	–0.731 **	0.067	–0.730 **
Potassium (mmol/L)	–0.583 *	–0.701 **	–0.061	–0.681 **
Calcium (mmol/L)	–0.505 *	–0.556 *	–0.153	–0.540 *
Magnesium (mmol/L)	–0.482	–0.464	–0.275	–0.454
Phosphorus (mmol/L)	–0.570 *	–0.621 *	–0.185	–0.614 *
Chlorine (mmol/L)	–0.559 *	–0.734 **	0.062	–0.733 **
Creatinine (µmol/L)	–0.468	–0.571 *	–0.034	–0.555 *
USG	–0.228	–0.236	–0.098	–0.240
pH	0.267	0.161	0.340	0.159
Serum				
Osmolality (mOsm/kg)	0.150	0.115	0.143	0.109
Sodium (mmol/L)	0.265	0.197	0.264	0.215
Potassium (mmol/L)	0.000	0.010	–0.020	0.021
Calcium (mmol/L)	0.109	0.216	–0.154	0.241
Magnesium (mmol/L)	0.408	0.347	0.325	0.362
Phosphorus (mmol/L)	0.191	0.138	0.199	0.139

TWI: total water intake; TFI: total fluid intake; MMDs: milk and milk derivatives; FVDs: fruit and vegetables drinks; BPDs: botanical protein drinks; SSDs: sugar-sweetened drinks; USG: urine specific gravity. The formula for calculating 24 h urine volume (mL) = 24 h urine weight (g)/USG. * *p* < 0.05; ** *p* < 0.01; *** *p* < 0.001.

## Data Availability

Data are contained within the article and Supplementary Materials.

## Supplementary Materials

The following supporting information can be downloaded at: https://www.mdpi.com/article/10.3390/nu16010116/s1, Figure S1: Flow chart of quality control; Table S1: The temperature and humidity of the environment.
