# Supplementary material for: Water Intake and Hydration Status among Pregnant Women in the Second Trimester in China: A Three-Day Metabolic Trial
Source: Nutrients. 2023 Dec 29;16(1):116. doi: 10.3390/nu16010116 (PMC10780576; doi:10.3390/nu16010116)
Supplement: Supplementary file 1 [file nutrients-16-00116-s001.zip › nutrients-2747422-supplementary.pdf]

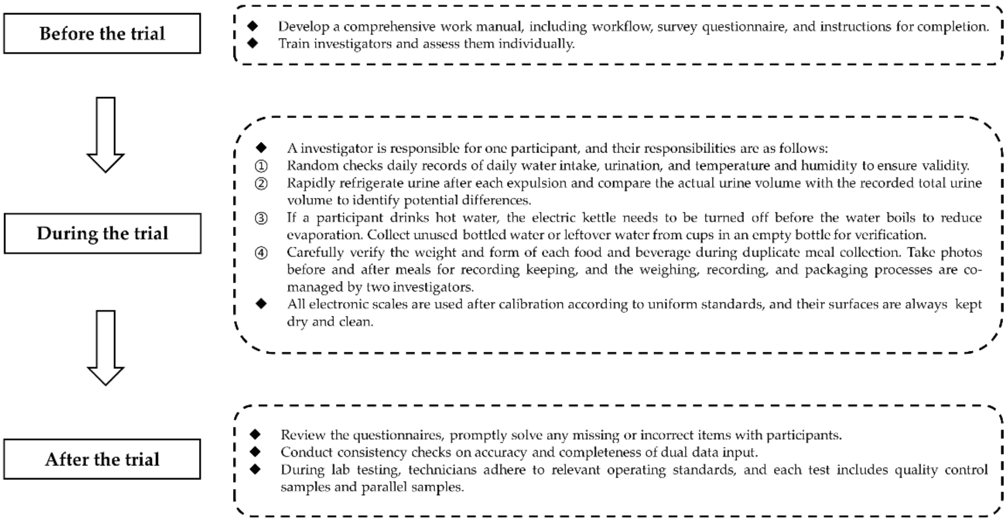

Figure S1. Flow chart of quality control

Table S1. The temperature and humidity of the environment

Variables	Indoor		Outdoor	
	Temperature (℃)	Humidity (%)	Temperature (℃)	Humidity (%)
**Day 1**	22.4	35	9.2	70
10:00 am	22.4	37	9.9	73
2:00 pm	22.2	34	9.7	69
8:00 pm	22.6	33	8.0	68
**Day 2**	21.9	33	6.9	69
10:00 am	21.6	34	7.0	76
2:00 pm	22.2	32	8.2	68
8:00 pm	21.9	33	5.5	63
**Day 3**	21.8	30	6.1	55
10:00 am	22.0	32	6.4	57
2:00 pm	21.6	29	7.4	51
8:00 pm	21.9	30	4.6	59
**Mean**	22.0	32	7.4	65
